# Supplementary material for: Precision digital mapping of endogenous and induced genomic DNA breaks by INDUCE-seq
Source: Nat Commun. 2022 Jul 9;13:3989. doi: 10.1038/s41467-022-31702-9 (PMC9271039; doi:10.1038/s41467-022-31702-9)
Supplement: Supplementary file 1 — Supplementary Information [file 41467_2022_31702_MOESM1_ESM.pdf]

Supplementary Information

Precision digital mapping of endogenous and induced genomic DNA breaks by INDUCE-seq

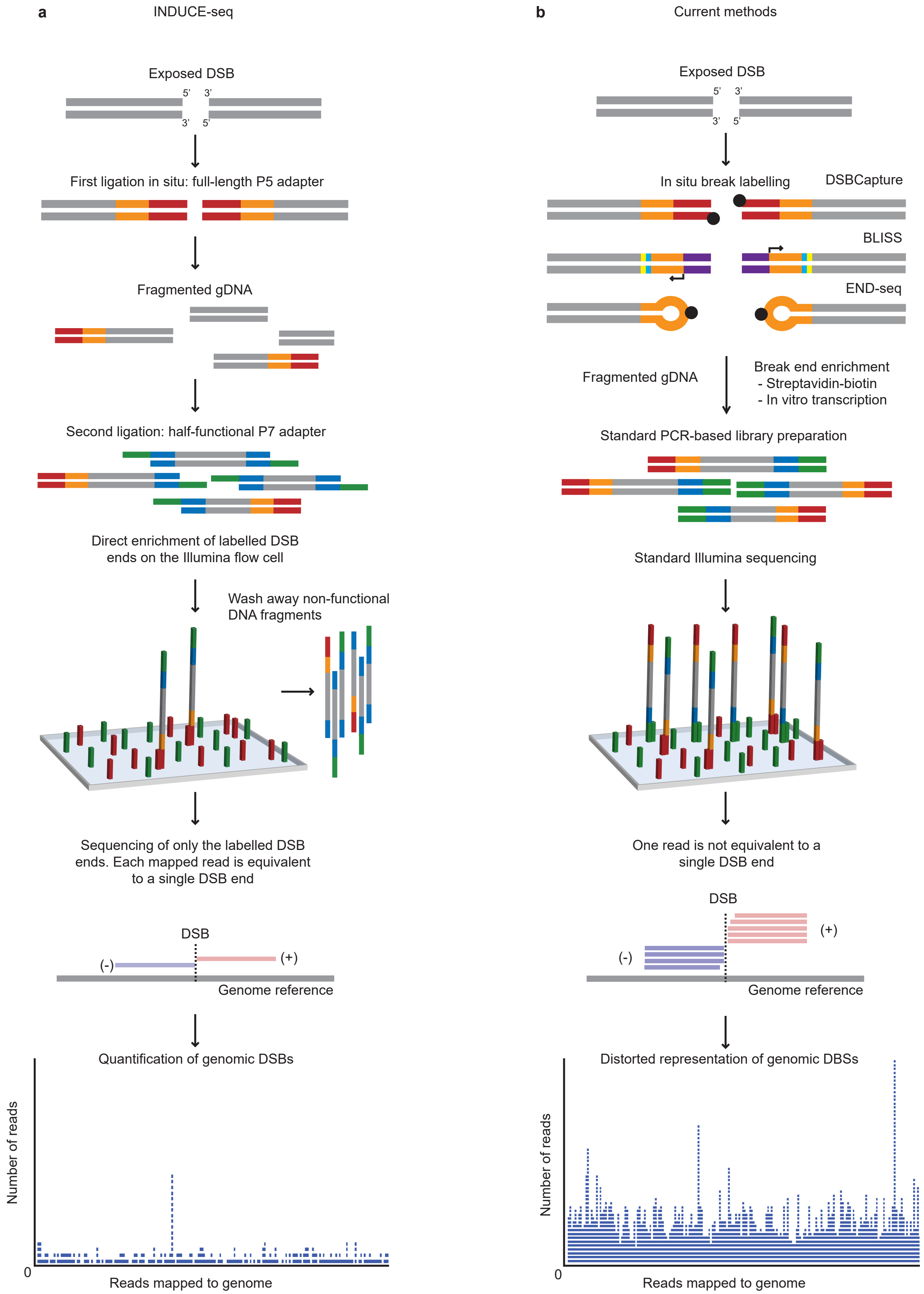

Supplementary Figure 1: Comparison of INDUCE-seq and current DSB mapping workflows. (a) Overview of INDUCE-seq workflow. The sequencing of INDUCE-seq libraries generates a quantitative output where one read is equivalent to one break. (b) Overview of the DSBCapture, BLISS and END-seq workflows. Sequencing following standard library construction generates an output where one read is not equivalent to a single DSB.

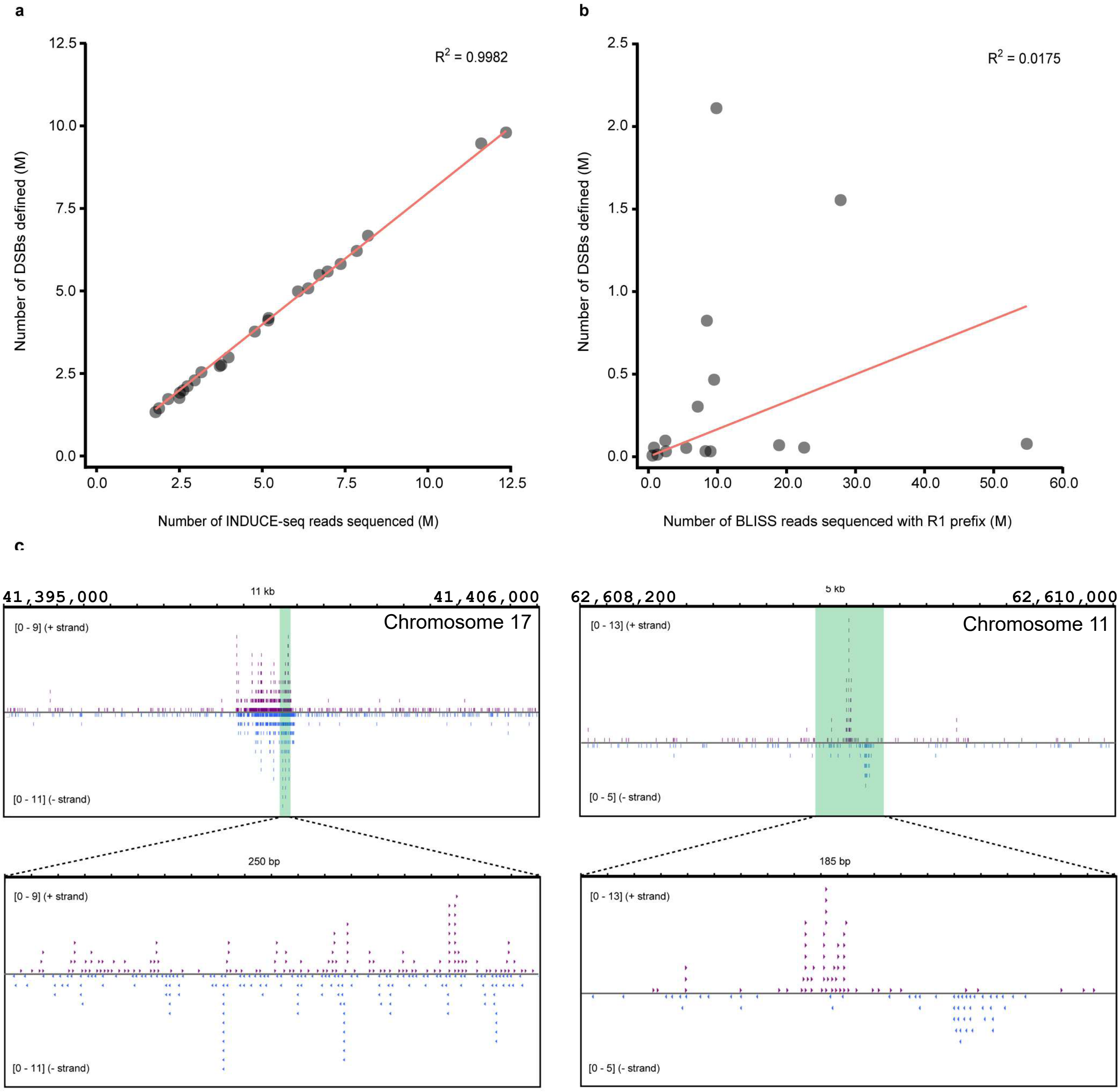

Supplementary Figure 2: Comparison between the number of reads sequenced and number of DSBs defined for INDUCE-seq and BLISS NGS libraries. (a) Scatter plot of the number of INDUCE-seq reads sequenced (millions) and the number of breaks defined (millions) from individual INDUCE-seq experiments. (b) Scatter plot showing the number of BLISS reads sequenced (millions) containing the R1 barcode prefix and the number of breaks defined (millions), following UMI error correction, from individual BLISS experiments. (c) Genome browser view of two recurrent endogenous DSB positions in HEK293 cells. (c, left panel) 11kb view of a DSB hotspot on chromosome 17. Purple arrows represent DSB ends labelled on the right side (+ strand) and blue arrows represent DSB ends labelled on the left side (- strand). Recurrent DSBs are evenly distributed throughout the hotspot region. (c, right panel) 2kb view of a chr11 DSB hotspot. Recurrent DSBs can be detected at different positions on the plus and minus strands. Source data are provided as a Source Data file.

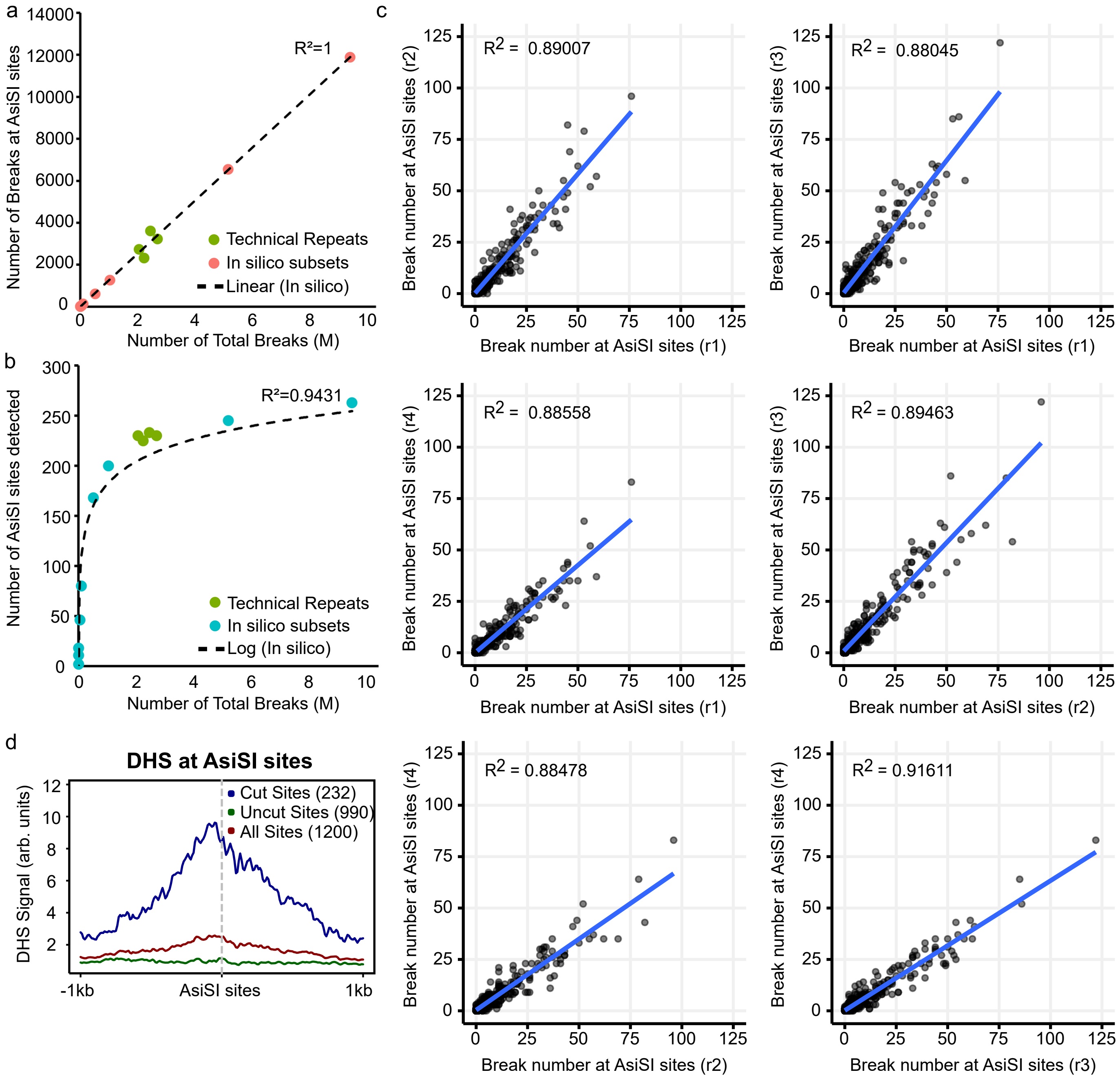

Supplementary Figure 3: Comparison of biological repeats demonstrates reproducibility of nuclease-induced DSB detection by INDUCE-seq. (a) The relationship between the number of breaks at AsiSI sites detected is linear when compared to the total number of breaks sequenced. (b) The relationship between the number of AsiSI sites detected is shown as a function of total number of breaks measured. The total number of AsiSI sites detected is highly reproducible between 4 technical repeats and following in silico subsetting. (c) Pairwise comparison of 4 technical repeat data sets demonstrating high reproducibility. (d) Composite plot showing DHS signal at difference classes of AsiSI sites. The DHS signal around INDUCE-seq-detected cut AsiSI sites (n=232) is shown in blue. The DHS signal around uncut AsiSI sites (n=990) is shown in green and around all AsiSI sites (n=1200) is shown in red. Source data are provided as a Source Data file.

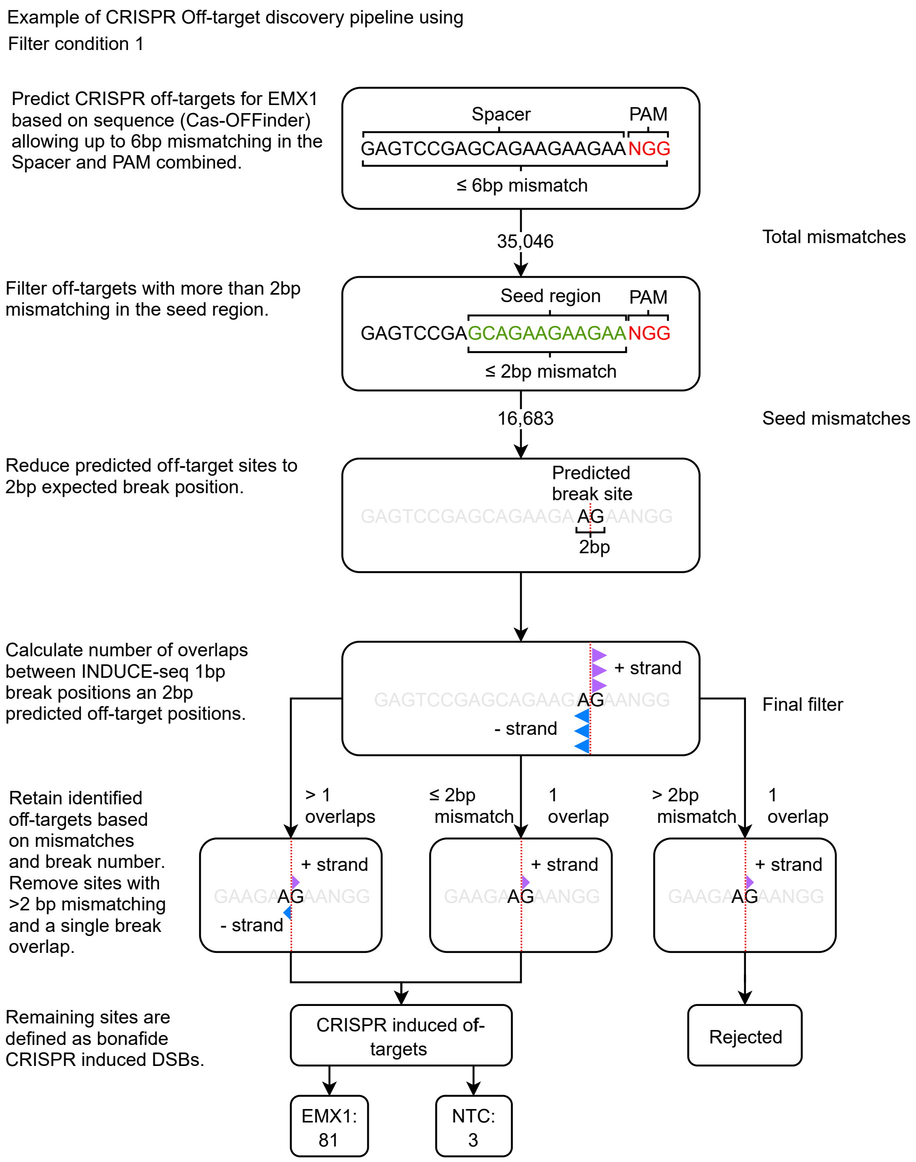

Supplementary Figure 4: CRISPR off-target discovery pipeline. Example of the procedure for the identification of off-target CRISPR-induced DSBs. The numbers shown refer to filter condition 1 in the procedure. NTC: Non-Treated Control.

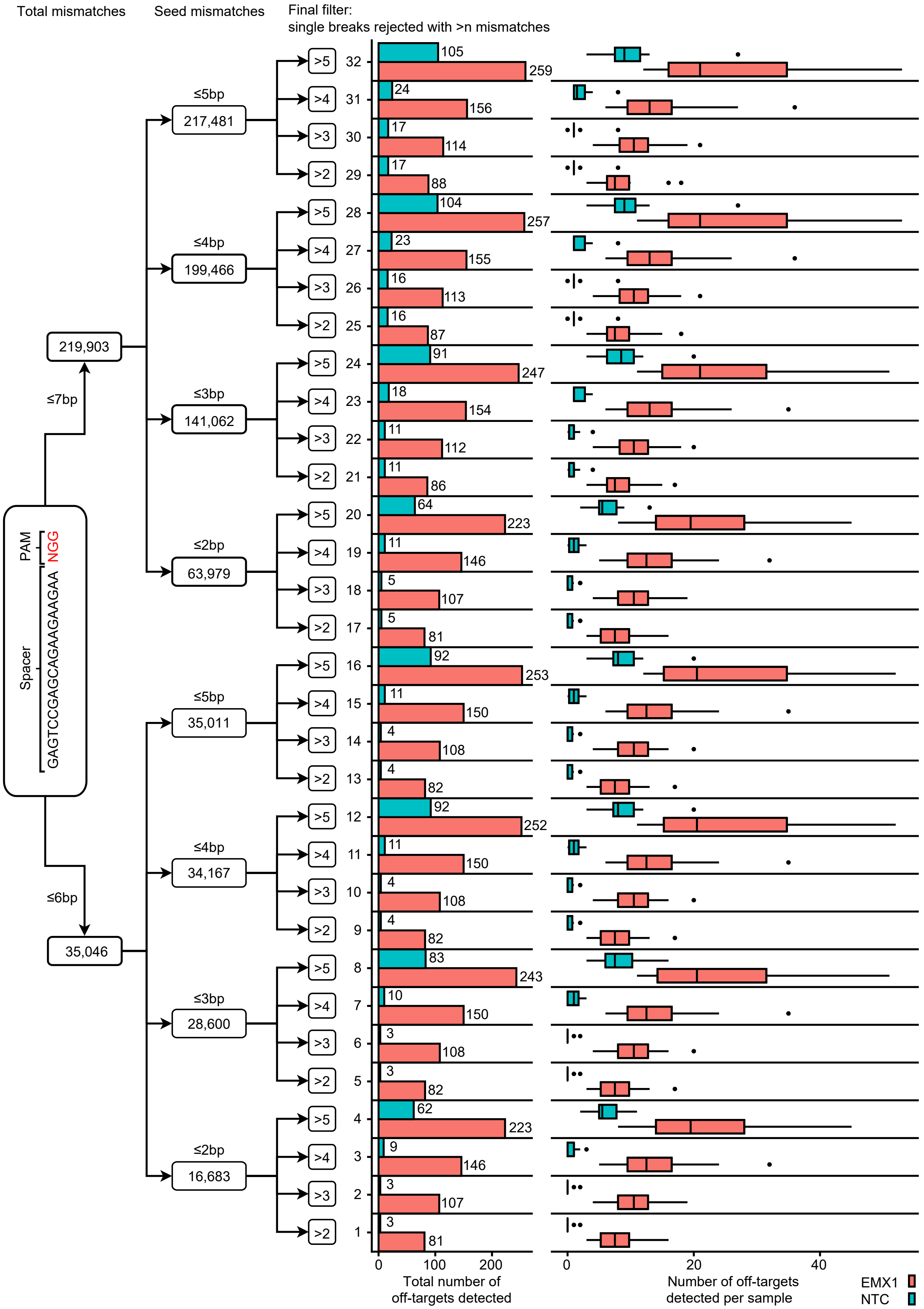

Supplementary Figure 5: Overview of all filter conditions used for off-target discovery. (Left side) The three levels of filtering employed as described in the discovery pipeline. A variable number of mismatches was tested at each level of filtering, resulting in 32 combinations of filter conditions. (Right side) For each filter condition a set of CRISPR-induced DSBs were derived from 5 experimental samples taken between 0h to 30h after treatment. Two biological experiments for EMX1 treated and control samples were conducted. The total number of off-target sites for all EMX1 and control samples combined are shown in the bar chart and listed. The boxplots show the distribution of the number off-targets detected for each individual dataset (n=5) from two independent biological experiments. The box plots indicate the minima, maxima, median, interquartile range at the 25 and 75 percentile (box) and the 5h and 95th percentile (whiskers). NTC: Non-Treated Control. Source data are provided as a Source Data file.

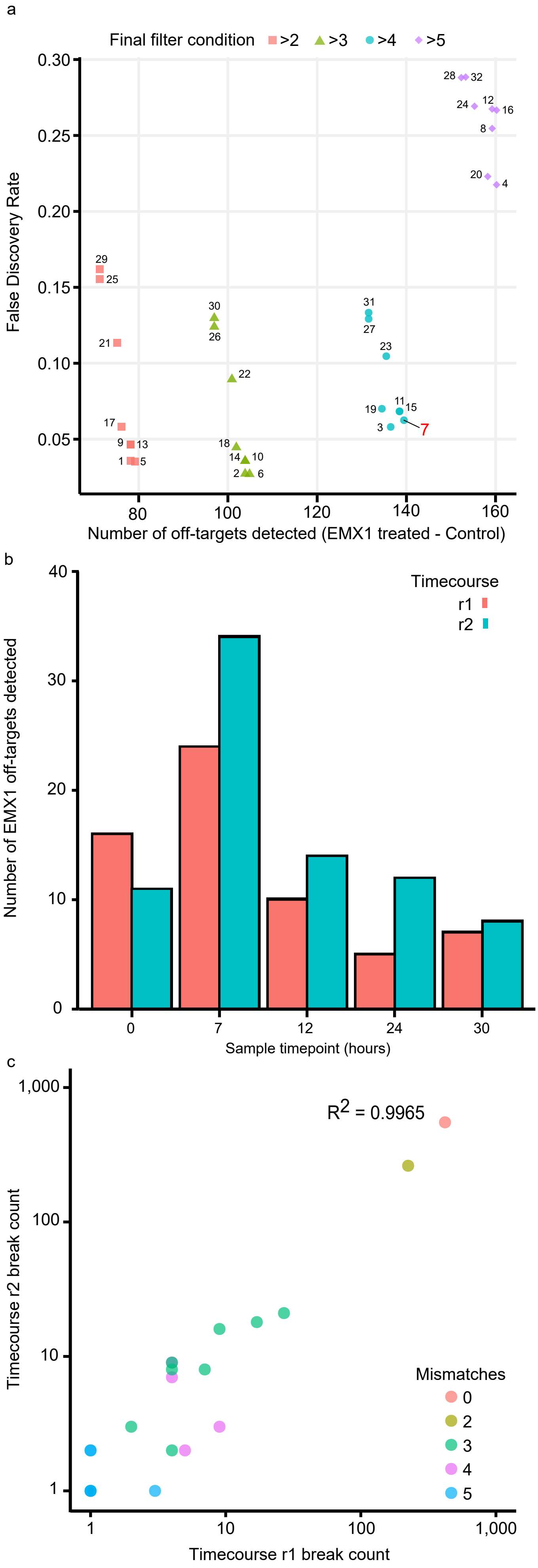

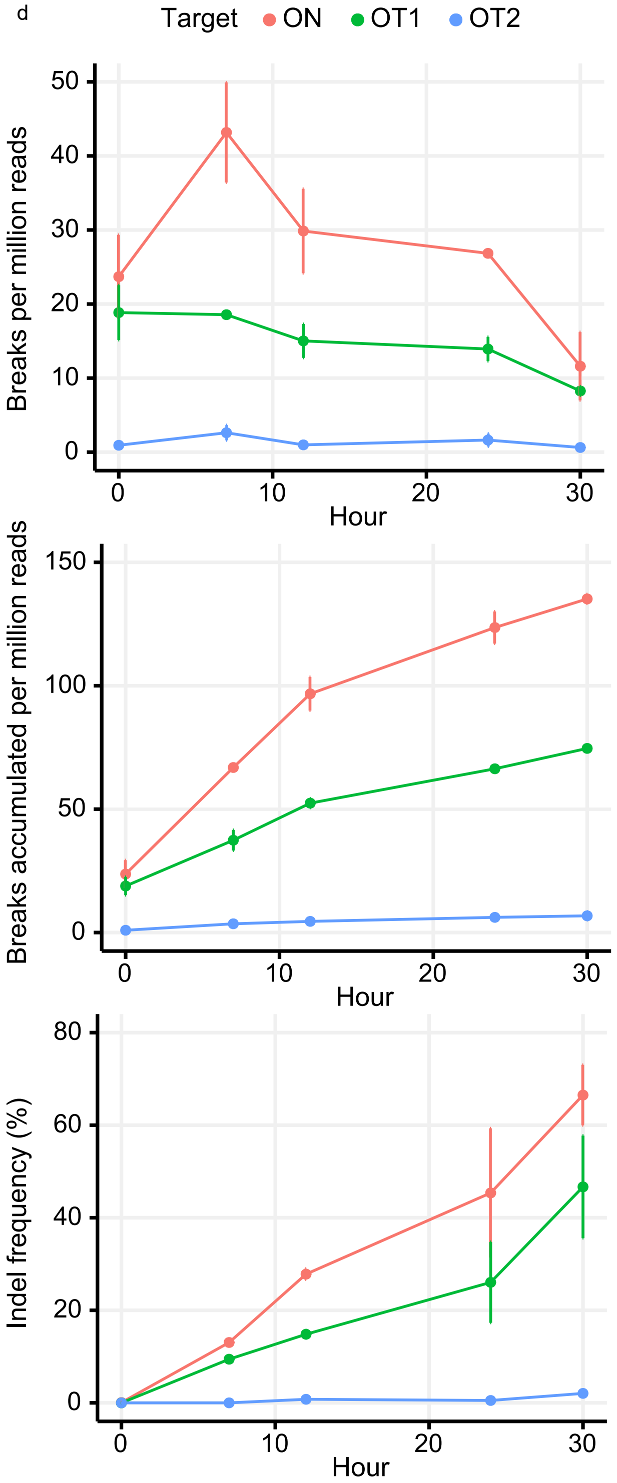

Supplementary Figure 6: Selection of parameters for determining CRISPR off-target discovery. (a) Scatterplot showing the relationship between EMX1 off-target site discovery and their FDR for each filter condition (1-32). Filter condition 7 (red highlight) was selected for all subsequent analysis. (b) Comparison between the number of EMX1 off-targets detected across r1 and r2 time course experiments. (c) Scatterplot showing the break number found at CRISPR off-target sites identified in two independent biological repeats. (d) Kinetics of editing across the multiple time points shown expressed as breaks per million reads (top panel), accumulated breaks per million reads (middle panel) and indel frequency (bottom panel). In each plot the on-target is highlighted in red, off-target number 1 is green and off-target number 2 is blue. Data presented as mean with a SD from 2 biological experiment. Source data are provided as a Source Data file.

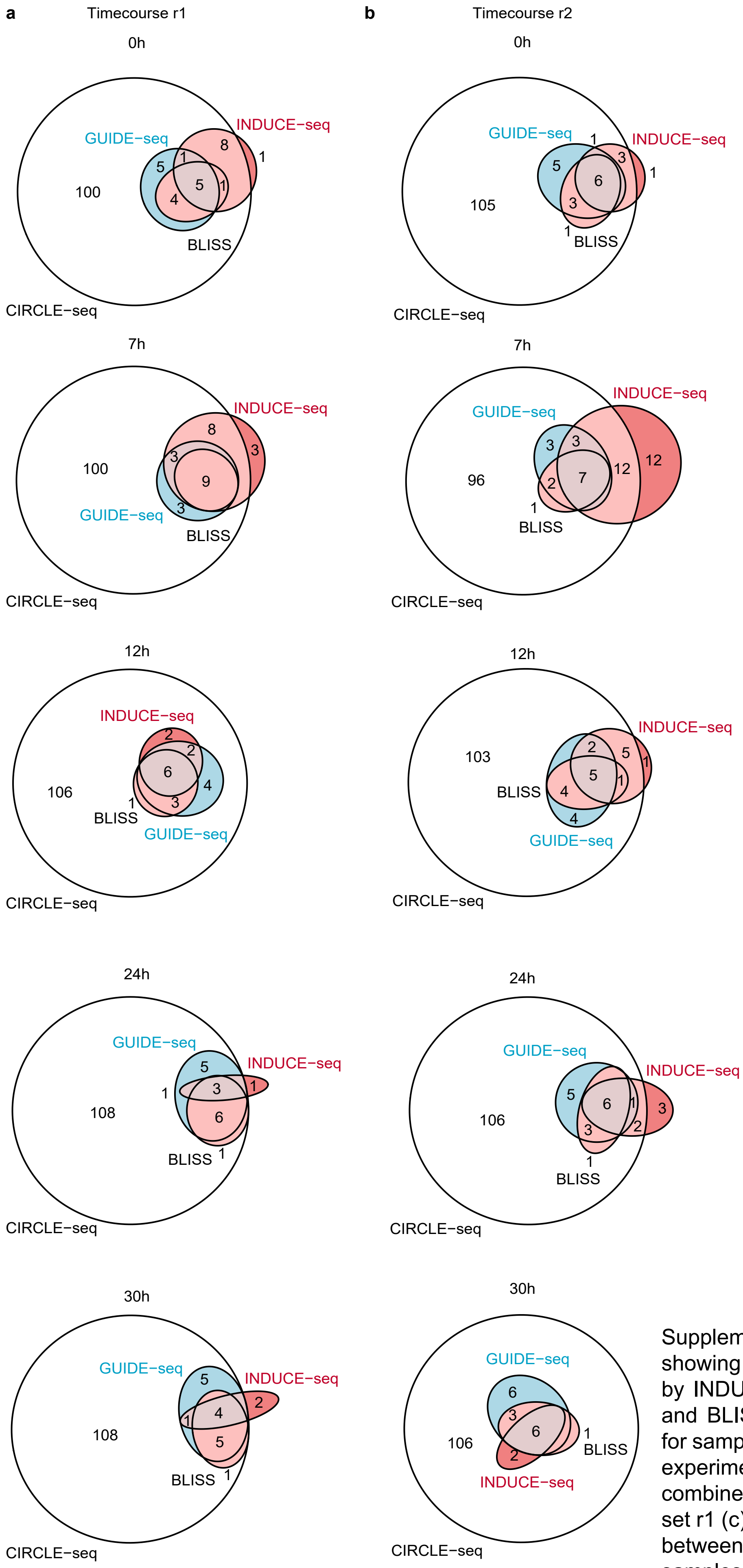

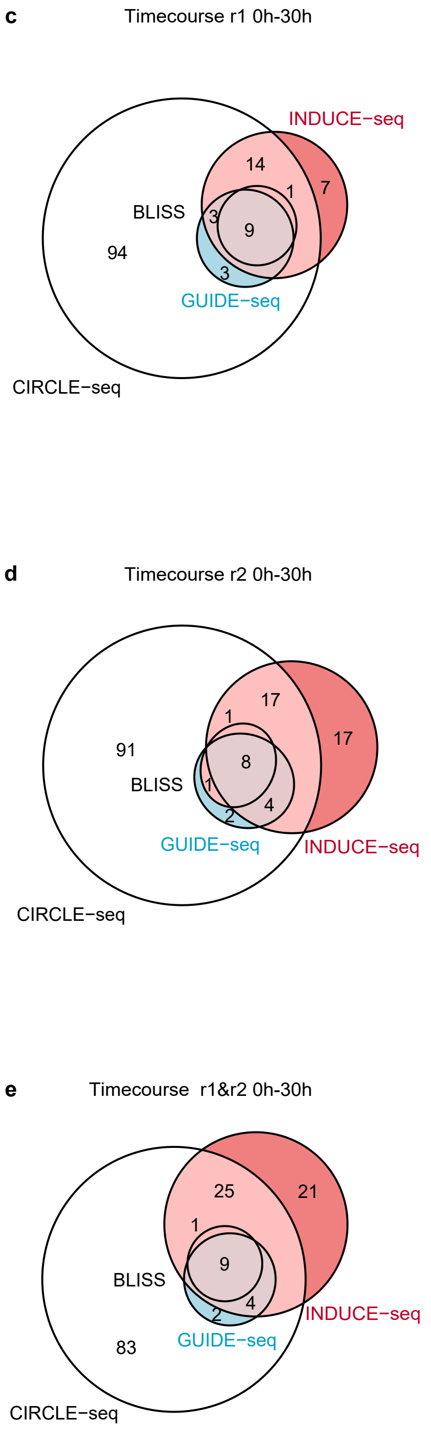

Supplementary Figure 7. Venn diagrams showing overlaps of the off-targets identified by INDUCE-seq, CIRCLE-seq, GUIDE-seq and BLISS. (a and b) Overlaps calculated for samples 0h to 30h from two independent experiments r1 (a) and r2 (b). (c and d) The combined overlaps from all time points for set r1 (c) and r2 (d). (e) Overlaps calculated between methods when all INDUCE-seq samples are combined.

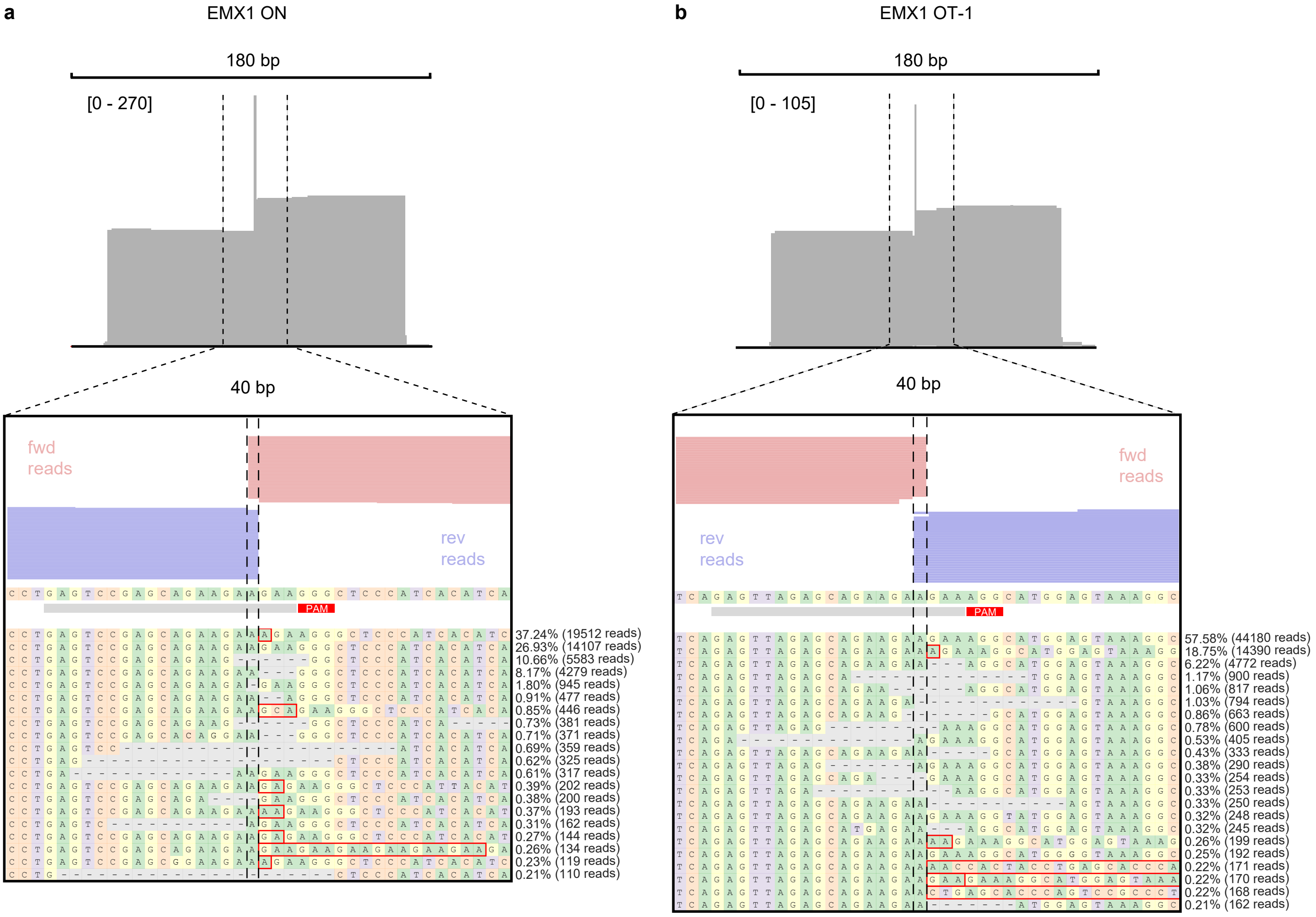

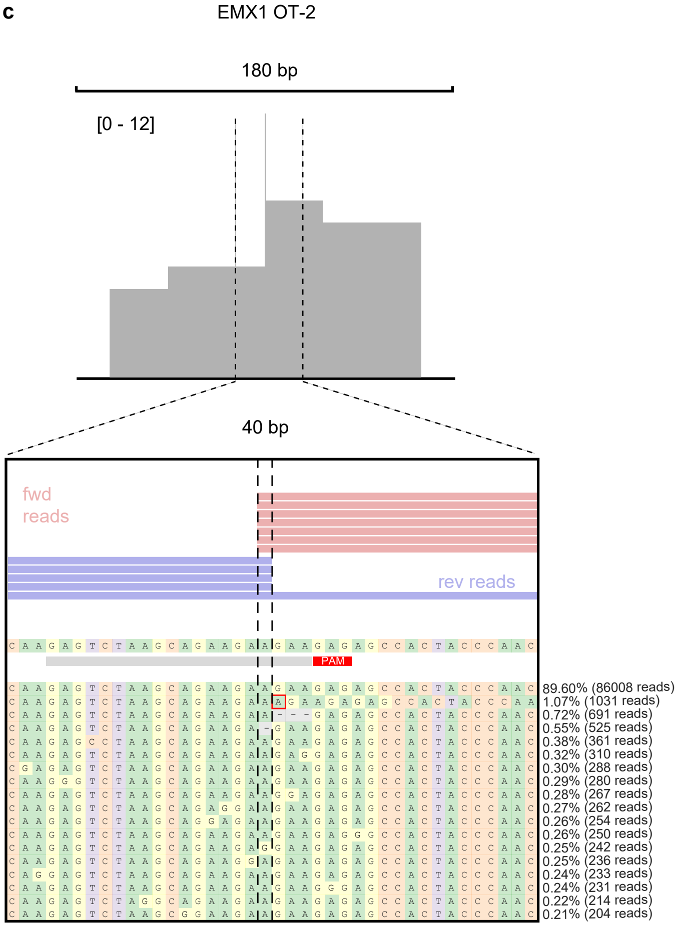

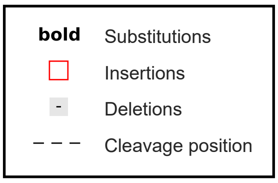

Supplementary Figure 8. The INDUCE-seq detected DSB pattern at CRISPR induced on- and off-target sites relates to editing outcome. Coverage tracks of the EMX1 on-target (a) and the two top ranking off-targets, OT-1 (b), and OT-2 (c), spanning 180bp. A close-up view of the 40bp region surrounding each target site, INDUCE-seq shows a distinct 1bp overhanging cleavage pattern rather than the usual Cas9-induced blunt DSB. Corresponding indel spectra, as measured by amplicon sequencing, at each site, shows the position of the indel mutations in relation to the observed break sites.
